# Xanthan gum enhances humoral immune response elicited by a DNA vaccine against leptospirosis in mice

**DOI:** 10.1186/1753-6561-8-S4-P153

**Published:** 2014-10-01

**Authors:** Marcelle Silveira, Samantha Vargas, Marcelo Mendonça, Carlos Eduardo Cunha, Daine Hartwig, Amilton Seixas, Suely Bampi, Amanda Rodrigues, Fabrício Conceição, Ângela Moreira

**Affiliations:** 1 Centro de Desenvolvimento Tecnológico, Núcleo de Biotecnologia, Universidade Federal de Pelotas, Brazil; 2 Instituto de Biologia, Departamento de microbiologia e parasitologia, Universidade Federal de Pelotas, Brazil; 3 Faculdade de Nutrição, Universidade Federal de Pelotas, Brazil

## Background

Traditional vaccines (killed or inactivated) played vital roles in controlling and eradicating infectious diseases for a long time. Antigen-specific T cell response can be induced when mice are intramuscularly inoculated with naked plasmid DNA. Therefore, DNA vaccines were evaluated in many studies, demonstrating its safety, stability and easy production. Furthermore, it has been reported that the gene expression lasted for one year after intramuscular injection of the plasmid DNA. However, some disadvantages such as the low transfection rate and low immunogenicity make the use of multiple doses necessary [1]. In light of this context, several studies have been performed to improve the immune response induced by DNA vaccines. The xanthan gum is an extracellular polysaccharide produced during fermentation of bacteria of the genus *Xanthomonas *and has been studied as a new vaccine adjuvant [2]. However, it has not yet been evaluated as an adjuvant for DNA vaccines. The aim of this study was to evaluate the capacity of the xanthan gum to increase the humoral immune response of mice inoculated with a DNA vaccine against a fragment of the leptospiral antigen LigAni cloned in the mammalian expression plasmid pTARGET.

## Material and methods

Four to six month-old female BALB/c mice were segregated into 2 groups of 12 animals each, where both groups were inoculated with the DNA vaccine. Animals belonging to group 2 (G2) also received 0.5% xanthan gum as adjuvant. The immunization protocol consisted of 3 doses (days 0, 14 and 21) of 100 µg of the pTARGET/*ligAni *plasmid injected intramuscularly 45 minutes after 100 μL of a 25% (w/v) sucrose solution was injected. Blood samples were taken 0, 13, 20 and 27 days post-first immunization for the humoral immune response evaluation after each dose through indirect ELISA using recombinant LigAni (5 µg.mL-1) as antigen. Sera were diluted 1:30 in PBS-T. ELISA units were calculated by dividing the mean absorbance of each group by the mean absorbance of the same group at day 0. ANOVA followed by the Tukey's test was performed to detect significant differences (p <0.05) between immunizations.

## Results

ELISA results showed that antibody titers in sera from G2 was statistically higher than titers in sera from G1 after the second and third immunization (p < 0,05). There was no significant difference between the second and third immunizations of G2 though. This way, the results indicate that the xanthan gum had an adjuvant.

## Conclusions

We conclude that xanthan gum had an adjuvant effect increasing the humoral immune response elicited by a DNA vaccine against leptospirosis in mice.
